# Real-time measurements of aminoglycoside effects on protein synthesis in live cells

**DOI:** 10.1073/pnas.2013315118

**Published:** 2021-02-22

**Authors:** Javier Aguirre Rivera, Jimmy Larsson, Ivan L. Volkov, A. Carolin Seefeldt, Suparna Sanyal, Magnus Johansson

**Affiliations:** ^a^Department of Cell and Molecular Biology, Uppsala University, 752 36 Uppsala, Sweden

**Keywords:** translation, antibiotics, single-molecule tracking, superresolution microscopy, tRNA

## Abstract

The spread of antibiotic resistance is turning many of the currently used antibiotics less effective against common infections. To address this public health challenge, it is critical to enhance our understanding of the mechanisms of action of these compounds. Aminoglycoside drugs bind the bacterial ribosome, and decades of results from in vitro biochemical and structural approaches suggest that these drugs disrupt protein synthesis by inhibiting the ribosome’s translocation on the messenger RNA, as well as by inducing miscoding errors. So far, however, we have sparse information about the dynamic effects of these compounds on protein synthesis inside the cell. In the present study, we measured the effect of the aminoglycosides apramycin, gentamicin, and paromomycin on ongoing protein synthesis directly in live *Escherichia coli* cells by tracking the binding of dye-labeled transfer RNAs to ribosomes. Our results suggest that the drugs slow down translation elongation two- to fourfold in general, and the number of elongation cycles per initiation event seems to decrease to the same extent. Hence, our results imply that none of the drugs used in this study cause severe inhibition of translocation.

## Significance

To address the public health challenges caused by the spread of antibiotic resistance, it is critical to enhance our understanding of the mechanisms of action of these compounds. In the present study, we use superresolved single-molecule tracking techniques, to investigate the effect of three aminoglycoside drugs on protein synthesis kinetics directly inside live bacterial cells. Our results imply that these drugs do not completely inhibit bacterial protein synthesis, but only make it slower. Hence, the bactericidal effect of these drugs is likely due to a disturbed, rather than inhibited, protein synthesis process.

Antibiotic resistance has become one of the biggest public health challenges of the 21st century. What used to be easily treatable diseases are becoming deadly as a consequence of commonly used antibiotics increasingly turning ineffective. To aid the development of new strategies to address this challenge, it is necessary to improve our understanding of the mechanism of action of these antibacterial compounds. Many antibiotics currently in use target the bacterial ribosome with high specificity (1). These compounds affect different stages of protein synthesis, depending on their binding sites in the bacterial ribosome or their binding to protein factors involved in protein synthesis.

Aminoglycosides are a class of natural and semisynthetic chemical compounds of broad-spectrum therapeutic relevance (2, 3) categorized as critically important by the World Health Organization (4). Aminoglycosides are presently used against multidrug-resistant bacterial infections (5, 6) and, more recently, considered as potential treatments for genetic diseases such as cystic fibrosis and Duchenne muscular dystrophy (3, 7, 8). The clinical relevance of aminoglycosides is only shadowed by side effects such as nephrotoxicity and irreversible ototoxicity (5, 6). A subclass of these molecules has a conserved aminocyclitol, a 2-deoxystreptamine, with linked amino sugar groups at different positions. Structural studies showed that these molecules bind at the major groove of the 16S ribosomal RNA (rRNA) in the A-site in close contact with the decoding center of the bacterial 30S ribosomal subunit (9–12). At the decoding center, the adenines A1492 and A1493 take part in monitoring the correct codon–anticodon interaction (13). Aminoglycoside molecules bound to this site have been suggested to interact with A1492/1493 and restrict their mobility (12, 14), which in turn interferes with the selection of cognate transfer RNA (tRNA) (9, 11, 15–18) as well as with the translocation step (11, 16, 19–22).

A secondary binding site for 4,5- and 4,6-substituted aminoglycosides has been identified at H69 in the 50S ribosomal subunit, in close contact with A- and P-site tRNAs (23). Based on crystal structures (23) and in vitro kinetics assays (24), it has been suggested that drugs bound to this secondary binding site affect ribosome recycling and also intersubunit rotation—potentially also affecting translocation.

The synergistic effect of aminoglycosides binding to multiple sites in the bacterial ribosome contributes to the misreading of codons and defective translocation, which eventually leads to cell death. The mechanism of action of various aminoglycosides on the ribosome has been characterized using diverse structure biology methods (as reviewed in ref. 25), classical in vitro functional biochemical assays (15, 20, 26), and, more recently, in vitro single-molecule approaches (11, 21, 27). Even though the mechanistic steps are described in detail by these complementary in vitro techniques, the reported effects of these drugs on the kinetics of protein synthesis are significantly different. For example, whereas single-molecule Förster resonance energy transfer (FRET) studies report a four- to sixfold inhibition of messenger RNA (mRNA) movement during translocation (21), stopped-flow experiments report a 160-fold inhibition (20). Recent advances in live-cell single-molecule tracking methods have now opened up the possibility to measure the drug’s effects on protein synthesis kinetics directly in live cells (28, 29).

In the present study, we measured the effect of three structurally different aminoglycosides, apramycin, gentamicin, and paromomycin, on the kinetics of translation elongation at a single-ribosome level in live *Escherichia coli* cells. By tracking single dye-labeled tRNAs and analyzing the diffusion trajectories using a Hidden Markov Model-based (HMM) approach, we measured dwell-times of elongator [Cy5]tRNA^Phe^ and initiator [Cy5]tRNA^fMet^ on the ribosome, which suggest an overall slower, but ongoing, protein synthesis in intact cells exposed to the aminoglycosides.

## Results

### Selection of Aminoglycoside Concentration Based on Growth Experiments.

To examine the effects of apramycin, gentamicin, and paromomycin on the kinetics of protein synthesis directly inside *E. coli* cells, we employed our recently developed single-tRNA tracking approach (28, 29). The labeled tRNAs actively take part in translation both in vitro and in vivo (28). A similar tracking approach has also been used by others to distinguish tRNA–ribosome bindings, although not for kinetics studies (30). To carry out the tracking experiments at an appropriate antibiotic concentration, we first measured the growth rate of *E. coli* cells in liquid rich-defined-medium (RDM) exposed to increasing aminoglycoside concentrations (0.001–100 µg/mL). With this assay, we found that the drugs completely inhibit bacterial growth at concentrations equal and higher than 10 µg/mL for apramycin, and above 1 µg/mL for gentamicin and paromomycin. These results agree with the reported minimum inhibitory concentration (MIC) of apramycin, gentamicin, and paromomycin in the range of 0.5–32 µg/mL (5, 14, 31–34).

We further explored the phenotypic effects of these antibiotics on single *E. coli* cells by following cell growth in a microfluidic device (35). We imaged growing cells in RDM for 1 h in ∼360 cell traps per experiment, then supplied 100 µg/mL of the respective antibiotic to cells in one side of the chip while keeping the remaining half in RDM as control. We then imaged the bacteria for 1 h before changing the media back to RDM without antibiotics. We observed that *E. coli* cells in 95–100% of the channels stopped growing after 15–20 min of treatment for all three antibiotics, while cells in ∼95% of the control channels grew normally. Following drug removal, we did not observe any channel where cells resumed growth within 6 h of imaging (Movie S1).

Based on the batch growth analysis and microfluidics single-cell growth experiments, we decided to measure the effect of two different concentrations (10 and 100 µg/mL) of aminoglycosides on protein synthesis kinetics in live cells. Based on reported dissociation constants of paromomycin, gentamicin, and apramycin to the h44-binding site on the ribosomes—0.2, 1.7, and 6.3 µM, respectively (36)—and a reported fourfold effect of efflux pumps on the intracellular aminoglycoside concentration (37), we estimate that the primary ribosome binding site will be 95, 75, and 44% occupied at the lower (10 µg/mL) drug concentration, and 99, 97, and 89% at the higher (100 µg/mL) concentrations for paromomycin-, gentamicin-, and apramycin-treated cells, respectively. However, these are uncertain numbers considering the lack of information on the effect of efflux pumps on all these specific aminoglycoside drugs and that the binding constants in all cases were estimated using rRNA model-loops of h44.

### Dynamic Binding of [Cy5]tRNA^Phe^ to Ribosomes in the Presence of Aminoglycosides.

**Fig. 1.**
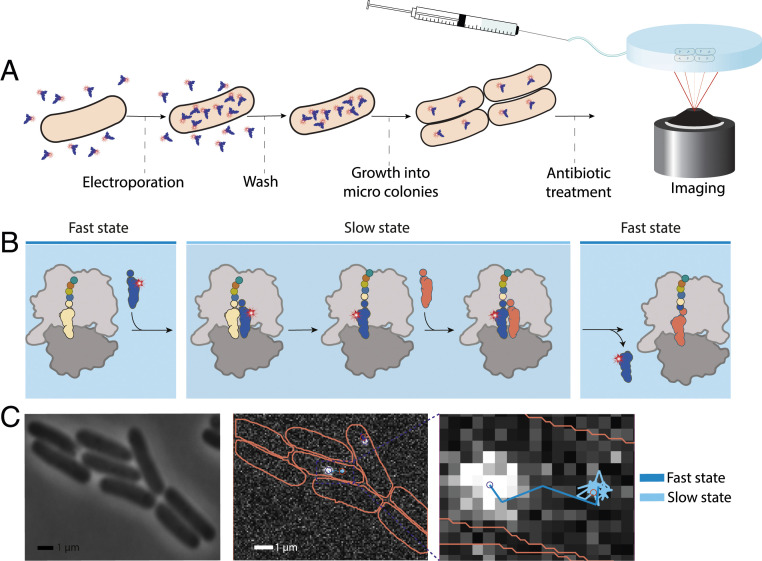
Electroporation and tracking of [Cy5]tRNA^Phe^ in aminoglycoside-treated *E. coli* cells (*A*) Microscopy sample preparation. (*B*) Representation of one [Cy5]tRNA^Phe^-binding cycle. The fast diffusion state is assigned to freely diffusing tRNA and tRNA bound to EF-Tu, while the slow state represents ribosome-bound tRNA, comprising two consecutive elongation cycles. (*C*) Example of segmented *E. coli* cells and the generated trajectories from the fluorescent particle tracking, fitted using HMM, and course-grained to two diffusional states—fast and slow.

To study how aminoglycosides affect translation elongation kinetics, we electroporated Phe-[Cy5]tRNA^Phe^ to live cells, then placed the bacteria on an agarose pad where the cells grew and divided for 1 h at 37 °C to form microcolonies (2–16 cells). After this time, we injected the respective aminoglycoside and incubated the cells under the microscope for an additional 1 h before acquiring fluorescence data (Fig. 1*A*). Within the time from the antibiotic treatment to the start of imaging (1 h), the *E. coli* cells in the agarose pads developed visible dense spots at both concentrations of all three drugs (*SI Appendix*, Fig. S1). We speculate that these spots are protein aggregates caused by erroneous or disrupted translation in the presence of the drugs, in line with previous microscopy studies of *E. coli* treated with the aminoglycoside streptomycin, which demonstrate visible protein aggregation (38).

After 1 h of drug treatment, we acquired bright-field, phase-contrast, and SYTOX blue images (405-nm laser illumination), in addition to fluorescence time-lapse movies (at 638-nm laser exposure) of diffusing [Cy5]tRNA^Phe^ from the microcolonies. Using our previously reported dot detection and tracking pipeline (28, 29), we detected the position of the fluorescent particles in all frames and built trajectories of their diffusion inside intact cells (not SYTOX blue stained). Cell contours were automatically assigned from the phase-contrast and bright-field images (see *Materials and Methods*). We processed the combined trajectories using an HMM analysis to estimate the frequency of transitions of tRNAs between different diffusive states for each dataset of 5,000–10,000 trajectory steps (*SI Appendix*, Fig. S2) and also using combined trajectories from all datasets of the same experimental condition (*SI Appendix*, Fig. S2). The best-fit model size, according to the Akaike Information Criterion (AIC), was coarse-grained down to two states—fast diffusion for free tRNA as well as tRNA bound to EF-Tu, and slow diffusion for ribosome-bound tRNA (Fig. 1*B*)—using a threshold of 1 µm^2^/s, as was done previously (28) (Fig. 1*C*). Our previous analysis of simulated microscopy data (28) showed that around 16,000 trajectory steps are enough to estimate the bound-state dwell-time with ∼10% SE. Hence, we expect that the bound-state dwell-time is rather poorly estimated in individual experiments, but that by combining the datasets (resulting in >25,000 trajectory steps), we should achieve reasonable convergence. The results from the individual experiments agree well with the results from the combined data (*SI Appendix*, Fig. S2).

**Fig. 2.**
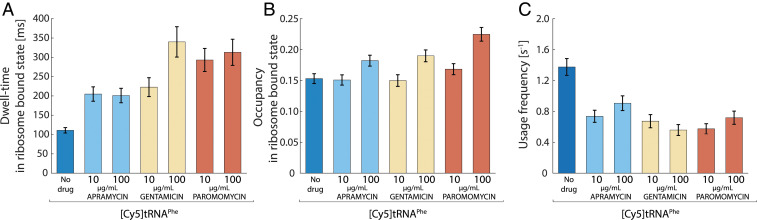
Longer and less frequent ribosome binding of [Cy5]tRNA^Phe^ during aminoglycoside treatment. Single-molecule tracking of Phe-[Cy5]tRNA^Phe^ in live *E. coli* cells, untreated or exposed to apramycin, gentamicin, and paromomycin at 10 and 100 µg/mL. Error bars represent bootstrap estimates of SEs. (*A*) HMM-estimated dwell-time of Phe-[Cy5]tRNA^Phe^ in the ribosome-bound state. (*B*) HMM-estimated occupancy of Phe-[Cy5]tRNA^Phe^ in the ribosome-bound state. (*C*) Estimated usage frequency of Phe-[Cy5]tRNA^Phe^ calculated from the occupancy in the ribosome-bound state divided by the corresponding dwell-time.

The average dwell-time of [Cy5]tRNA^Phe^ on ribosomes in the absence of any drug is around 100 ms (111 ± 7 ms), as reported previously (28). If we assume rapid dissociation of deacylated tRNA from the ribosomal E-site (39), this result suggests an average translation elongation cycle time of 56 ms per codon, in line with previous indirect in vivo estimates (40). In the presence of either of the three aminoglycosides tested, the dwell-time of [Cy5]tRNA^Phe^ on ribosomes increased two- to fourfold (Fig. 2*A* and *SI Appendix*, Table S1), suggesting a slowdown of translation elongation with the corresponding factor. While the bound-state dwell-time was the same at both drug concentrations in the case of apramycin and paromomycin (∼200 and ∼300 ms, respectively), the dwell-time increased from 220 ± 20 ms to 340 ± 40 ms for the corresponding change (10–100 µg/mL) in gentamicin concentration (Fig. 2*A*).

The HMM-estimated occupancy of [Cy5]tRNA^Phe^ in the slow diffusional state—a measure of the momentary fraction of [Cy5]tRNA^Phe^ bound to ribosomes at any given time (steady-state fraction)—was only slightly affected, with an ∼50% increase in the presence of 100 µg/mL paromomycin as the most significant deviation (Fig. 2*B* and *SI Appendix*, Table S1). All three antibiotics result in a slight increase in slow-state occupancy of [Cy5]tRNA^Phe^ at 100 µg/mL concentration compared to 10 µg/mL concentration. It should be noted that our previous experiments with [Cy5]tRNA^Phe^ tracking in rifampicin-treated cells resulted in practically complete abolishment (1%) of slow-state occupancy of [Cy5]tRNA^Phe^ (28). Moreover, when *E. coli* cells harboring aminoglycoside-resistant ribosomes (41) were treated with 100 µg/mL gentamicin, which showed the largest effect on [Cy5]tRNA^Phe^ dwell-time on wild-type ribosomes, no marked effect of the drug on [Cy5]tRNA^Phe^ diffusion was observed (*SI Appendix*, Fig. S3). The slow-state occupancy was 15 and 17%, and the slow-state dwell-time was 144 ± 7 ms and 154 ± 8 ms, in the absence or presence of gentamicin, respectively. These results thus nullify the possibility that the slow diffusion state of [Cy5]tRNA^Phe^, in the presence and absence of the aminoglycosides, arises from nonspecific binding of the [Cy5]tRNA^Phe^ to unrelated cellular elements and rather confirm that this state represents the binding of the labeled tRNAs to the ribosomes.

Overall, by tracking the binding of [Cy5]tRNA^Phe^ to ribosomes in aminoglycoside-treated cells, we find that the average bound-state dwell-time of [Cy5]tRNA^Phe^ increases, but that the steady-state fraction of [Cy5]tRNA^Phe^ bound to ribosomes is only slightly affected. We can further use the bound-state dwell-time and occupancy to calculate the frequency of usage of [Cy5]tRNA^Phe^ per cell. This calculation shows that in the presence of drugs, the frequency of [Cy5]tRNA^Phe^ usage decreases to ∼50% compared to that in untreated cells (Fig. 2*C*). If we assume that the total number of competing nonfluorescent tRNA^Phe^ inside the cells is the same before and after drug treatment, this result thus suggests that the number of amino acids incorporated into polypeptides per time unit per cell decreases only by a factor of 2, on average, in these severely growth-inhibited cells.

An alternative explanation to our results, more in line with previous findings of significant translocation inhibition by aminoglycoside drugs (20), could be that the dwell-time measurements of ribosome-bound tRNAs represent tRNA^Phe^ bindings only to the initial codons of the mRNAs, followed by a rather rapid dissociation of the resulting fMet-Phe-[Cy5]tRNA^Phe^ dipeptidyl-tRNA. To investigate this scenario further, additional experiments were performed.

### The Effect of Apramycin, Gentamicin, and Paromomycin on the First Elongation Cycle of a Translation Event.

The data presented so far from [Cy5]tRNA^Phe^ tracking represent average effects on all tRNA^Phe^ readings throughout the complete transcriptome. To specifically investigate the effect of the drugs on the initial events of translation, we repeated the experiments presented in the previous section, but now we electroporated and tracked Cy5-labeled initiator tRNA^fMet^. In this case, the dwell-time of the labeled tRNA in the slow diffusion state represents the time required to finish translation initiation, i.e., joining of the 50S ribosomal subunit, plus the duration of the first elongation cycle. If, however, translocation is entirely blocked by the presence of the aminoglycosides and, as discussed in the previous section, [Cy5]tRNA^Phe^ dissociates as dipeptidyl-[Cy5]tRNA^Phe^, the dwell-time of [Cy5]tRNA^fMet^ on ribosomes would instead represent the time required for subunit joining, the dissociation of fMet-aa-tRNA, plus the time required for the cell to rescue the stalled ribosome with deacylated [Cy5]tRNA^fMet^ in the P-site, i.e., most likely EF-G/RRF–based ribosome splitting. The latter number is, to our understanding, unknown in the context of the living cell, although estimates based on in vitro experiments suggest ∼200 ms per splitting event (42). Overall, however, the discussed scenario of complete translocation inhibition would result in considerably longer bound-state dwell-times for [Cy5]tRNA^fMet^ than for [Cy5]tRNA^Phe^.

**Fig. 3.**
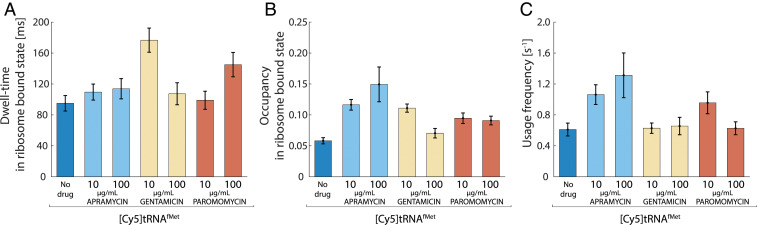
Measurements of the effect of aminoglycosides in the first elongation cycle. Single-molecule tracking of fMet-[Cy5]tRNA^fMet^ in live *E. coli* cells, untreated or exposed to aminoglycoside antibiotics at 10 and 100 µg/mL Error bars represent bootstrap estimates of SEs. (*A*) HMM-estimated dwell-time of fMet-[Cy5]tRNA^fMet^ in the ribosome-bound state. (*B*) HMM-estimated occupancy of fMet-[Cy5]tRNA^fMet^ in the ribosome-bound state. (*C*) Usage frequency of fMet-[Cy5]tRNA^fMet^ calculated using the dwell-times and occupancies at the ribosome-bound state.

From the HMM-estimated transition frequencies of [Cy5]tRNA^fMet^ between different diffusion states, we find that treatment with either of the three aminoglycosides results in longer or equal dwells of [Cy5]tRNA^fMet^ on ribosomes compared to in untreated cells (Fig. 3*A* and *SI Appendix*, Table S1), as well as a roughly proportional increase in the bound-state occupancy (Fig. 3*B* and *SI Appendix*, Table S1). For gentamicin- and paromomycin-treated cells, this results in an overall unchanged frequency of usage of [Cy5]tRNA^fMet^, whereas in apramycin-treated cells, [Cy5]tRNA^fMet^ is used approximately two times more often than in untreated cells (Fig. 3*C*). Importantly, the bound-state dwell-times of [Cy5]tRNA^fMet^ (Fig. 3*A*) are in all conditions shorter than the corresponding bound-state dwell-times of [Cy5]tRNA^Phe^ (Fig. 2*A*). Hence, these results together strongly suggest that the [Cy5]tRNA^Phe^-binding events observed do represent productive usage of Phe-[Cy5]tRNA^Phe^ on ribosomes, with proper transitions of the tRNA through the ribosomal tRNA-binding sites, although slowed down by the aminoglycoside drugs.

**Fig. 4.**
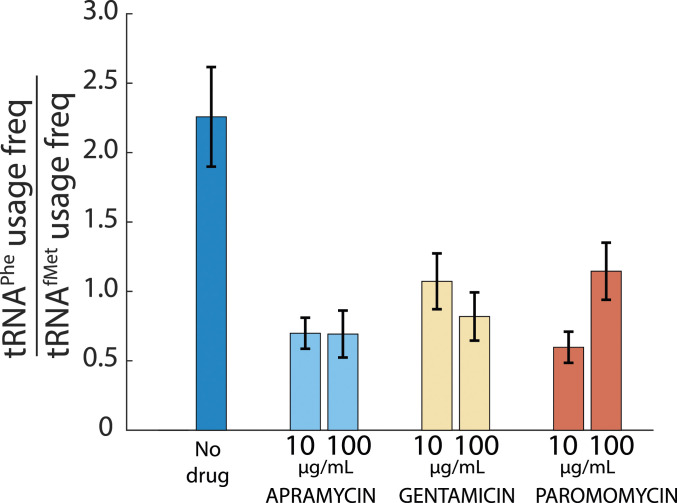
The [Cy5]tRNA^Phe^ usage relative to [Cy5]tRNA^fMet^ usage decreases in the presence of aminoglycosides. The usage frequency of [Cy5]tRNA^Phe^ relative to [Cy5]tRNA^fMet^ from cells exposed to aminoglycosides at 10 and 100 µg/mL The error bars represent calculated SE of the ratio, propagated from bootstrapped estimates of SEs of measured parameters.

When comparing the frequency of usage of [Cy5]tRNA^fMet^ (Fig. 3*C*) to that of [Cy5]tRNA^Phe^ (Fig. 2*C*), we find that all aminoglycosides tested result in a reduction of [Cy5]tRNA^Phe^ ribosome-binding events relative to [Cy5]tRNA^fMet^ (Fig. 4), suggesting relatively fewer elongation cycles per initiation event in drug-treated cells.

### Aminoglycosides Generally Affect the First Elongation Cycle Less than an Average Elongation Cycle.

**Fig. 5.**
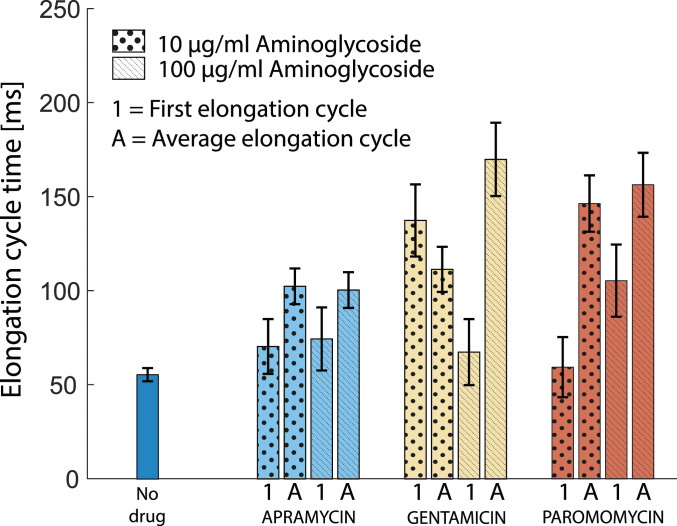
The impact of aminoglycosides in the first elongation cycle compared to an average elongation cycle. The first elongation cycle time was obtained by subtracting the subunit joining time (40 ms) calculated from [Cy5]tRNA^fMet^ tracking in the absence of antibiotics from the [Cy5]tRNA^fMet^ ribosome-bound dwell-time in cells exposed to the aminoglycosides. The average elongation time was calculated considering that the observed dwell-time of [Cy5]tRNA^Phe^ in the ribosome-bound state spans two elongation cycles at any point in the mRNA. Error bars represent bootstrap estimates of SEs propagated from the dwell-time error-estimates.

Finally, under the assumption that both [Cy5]tRNA^Phe^ and [Cy5]tRNA^fMet^ dwell-times in the slow diffusion state represent productive binding to ribosomes, we can use the HMM-estimated slow-state dwell-times to compare the effect of the drugs on the first elongation cycle of translation relative to the average elongation cycle. In the absence of drugs, the time to finish initiation once tRNA^fMet^ has bound can be calculated from the ribosome-bound dwell-time of [Cy5]tRNA^fMet^, 95 ± 10 ms (Fig. 3*A*), minus the time for an average elongation cycle, 55 ± 4 ms (Fig. 2*A*). This results in an estimated time for subunit joining of around 40 ± 11 ms. If we assume that the aminoglycosides do not interfere with the final steps of translation initiation (43), we can now subtract the time for these steps, 40 ± 11 ms, from the total dwell-time of [Cy5]tRNA^fMet^ in the presence of the drugs and achieve an estimate of the average time required for the very first elongation cycle on all mRNAs when exposed to either of the drugs. As can be seen in Fig. 5, the resulting estimate of the time required for the first elongation cycle is lower or similar to that of an average elongation cycle estimated from [Cy5]tRNA^Phe^ ribosome dwell-times in the presence of either of the drugs.

## Discussion

In this study, we directly measured the impact of three aminoglycoside drugs on the kinetics of protein synthesis in live bacterial cells. We tracked fluorescently labeled tRNA and used two critical parameters, tRNA dwell-time on ribosomes and the momentary occupancy of tRNA on ribosomes, to monitor the overall state of translation across all native mRNAs at physiological conditions. The application of this method could potentially become a shortcut to characterize the effects of different chemical compounds on protein synthesis directly inside bacterial cells.

A limitation in our method is that we cannot unambiguously distinguish productive ribosome bindings from unproductive or unspecific binding events based on the signal itself. However, in our previous study, we have shown that the bound-state dwell-time of [Cy5]tRNA^Phe^ gets longer when the experiment is performed in a strain harboring slow ribosomes (28). In the present study, we find that the bound-state dwell-time of [Cy5]tRNA^Phe^ in an aminoglycoside-resistant strain remained the same when the cells were treated with gentamicin. These observations strongly suggest that the slow diffusion state, irrespective of the treatment with aminoglycosides, do represent ribosome bindings. Further, the fact that the bound-state dwell-time of [Cy5]tRNA^Phe^ is longer than that of [Cy5]tRNA^fMet^ in drug-treated cells also suggests that the ribosomes must proceed beyond the first codon, as we have no evidence suggesting that P-site–bound tRNA^fMet^ would dissociate before translocation. Hence, we interpret the dynamic transitions of the labeled tRNAs into a slow diffusion state as mainly productive ribosome-binding events.

While treating *E. coli* cells with aminoglycosides, we measured longer dwells of [Cy5]tRNA^Phe^ and longer or equal dwells of [Cy5]tRNA^fMet^ on ribosomes, suggesting slower but still ongoing protein synthesis. The usage frequencies of [Cy5]tRNA^Phe^ and [Cy5]tRNA^fMet^ indicate that even though the number of initiation events per time unit per cell appears to be practically unchanged (Fig. 3*C*), the corresponding number of elongation cycles per time unit per cell was reduced roughly twofold (Fig. 2*C*).

Our measurements revealed two- to fourfold longer ribosome-bound [Cy5]tRNA^Phe^ dwell-times in cells exposed to either of the three aminoglycosides. These results agree, reasonably well, with previous in vitro single-molecule studies reporting a two- to sixfold slowdown of translocation rates for gentamicin and paromomycin (21) or lengthening of the rotated ribosome state for apramycin and paromomycin (11) and nonrotated state for gentamicin (11). We note, however, that the timescale of elongation cycles measured in vivo at 37 °C in this work is significantly different from that of elongation cycles measured at room temperature in the in vitro single-molecule fluorescence assays (11, 21).

The near-complete inhibition of translocation by paromomycin as reported previously from ensemble experiments in a reconstituted system (20) is difficult to reconcile with our present findings. We note that those experiments were performed with tRNA^Phe^ from yeast, whereas other components were from *E. coli*, which can be a potential reason. Other ensemble in vitro kinetics experiments, following tripeptide formation using native *E. coli* tRNAs (11), show slow but significant translocation in the presence of all three drugs, with a reduction in translocation rate somewhere in between those of the in vitro single-molecule experiments (11, 21) and the in vitro ensemble experiments (20). Finally, previous indirect reporter-based in vivo results (44) also suggest only partial inhibition of translation in the presence of the three aminoglycoside drugs in question at similar concentrations, with apramycin giving the weakest response, in line with our findings (Fig. 5).

If we assume that aminoglycosides do not cause any delay in 50S subunit joining, the comparison between tRNA^Phe^ and tRNA^fMet^ dwell-times on ribosomes (Fig. 5) shows that an average elongation anywhere in the mRNA is slower or similar to the first cycle when the cells are treated with either of the drugs. For gentamicin and paromomycin, we observe slightly different effects on the first elongation cycle relative to the average elongation cycle depending on the concentration of the drug (Fig. 5), which might be explained by limited accessibility of the drug to the secondary binding site during the first elongation cycle, as has been suggested previously (27).

Interestingly, while the usage frequency of [Cy5]tRNA^Phe^ decreased roughly twofold (Fig. 2*C*), in the presence of either of the three drugs at both concentrations, the usage frequency of [Cy5]tRNA^fMet^ remained the same (gentamicin and paromomycin) or increased (apramycin) (Fig. 3*C*). Since the usage frequency changes differently for tRNA^Phe^ and tRNA^fMet^, we do not think these results are secondary effects due to e.g., drug-induced up- or down-regulation of tRNA expression. Hence, we interpret this effect as a reduction in elongation cycles per initiation event (Fig. 4), which in turn could be due to premature termination caused by enhanced levels of misreading or frameshifting, or due to rapid recycling of translocation-inhibited ribosomes following drop-off of the peptidyl tRNAs from the ribosomal A-site. This result is in line with our initial speculation that the observed dense spots accumulating in aminoglycoside-treated cells (*SI Appendix*, Fig. S1) represent the aggregation of short peptides and/or misfolded proteins caused by tRNA misreading.

Recent in vitro experiments suggested that the aminoglycosides gentamicin and paromomycin perturb the ribosome recycling process by inhibiting tRNA release from posttermination complexes (22, 24). Our findings of increased or similar [Cy5]tRNA^fMet^ usage frequency in cells exposed to these aminoglycosides (Fig. 3*C*), suggesting the same or an increase in the number of initiations per time unit per cell, do not support such conclusions, as a depleting pool of available ribosomes would, most likely, cause a decrease of tRNA^fMet^ usage frequency. In conjunction with the two- to fourfold extended elongation cycle time (Fig. 2*A*) and a twofold decrease of tRNA^Phe^ usage frequency (Fig. 2*C*), these results overall suggest that the detrimental effect of these drugs on bacterial cells is not due to complete inhibition of translation, but can probably be attributed to a combination of their varying error-inducing capacity (as hypothesized in refs. 45 and 46) and their ability to slow down translocation, possibly with occasional peptidyl–tRNA drop-off events, resulting in erroneous and/or truncated polypeptides.

## Materials and Methods

### Reagents.

Stock solutions of apramycin (Sigma), gentamicin (Sigma), and paromomycin (Sigma) used at manufacturer’s purity (≥ 97%) were prepared in MiliQ water and had a final concentration of 25 mg/mL.

### Growth Rates.

*E. coli* DH5α cells (Invitrogen) were grown overnight at 37 °C in 5 mL of EZ RDM (Teknova). In a 100-well plate (honeycomb 2), wells were filled with 300 µL of RDM containing: no antibiotic, 0.001, 0.01, 0.1, 1, 10, and 100 µg/mL of antibiotic (apramycin, gentamicin, and paromomycin). From the overnight culture, 0.5 µL was added to each well, except for the wells used as blanks, and the optical densities at 600 nm were measured in a microplate reader (Bioscreen C, Oy) over 24 h at 37 °C with continuous orbital shaking. Each condition was performed in technical triplicates.

### Microfluidic Growth Experiments.

*E. coli* DH5α cells (Invitrogen) were inoculated into 5 mL of RDM and grown overnight at 37 °C with 200 rpm shaking. The overnight culture was diluted 1:500 in RDM supplemented with 0.0425% (wt/vol) Pluronic F108 (Sigma) and loaded into a polydimethylsiloxane (PDMS) microfluidic chip at 37 °C as described in ref. 47. The chip was designed with two independent sets of 1,500-nm-wide cell traps. The cells were allowed to grow in the microfluidic device for 1 h, taking phase-contrast images at 1-min intervals. After 1 h, cells on one side of the chip were supplied with RDM+pluronic with the respective antibiotic at 100 µg/mL, while cells on the second side were supplied only with RDM+pluronic. Phase-contrast images were taken at 1-min intervals from the channels exposed to antibiotics as well as from the control traps without antibiotics. After this time, all the channels were supplied with fresh RDM without antibiotics, and phase-contrast images were taken at 1-min intervals for 6 h.

The quantification was done by counting the number of channels containing growing cells before antibiotic treatment, the number of channels with growing cells that stopped growth after antibiotic treatment, and the number of channels with cells that resumed growth after supplying fresh RDM within 6 h.

### Preparation of *E. coli* Cells with Aminoglycoside-Resistant Ribosomes.

The A1408G point mutation in the 16S rRNA confers ribosome resistance against aminoglycoside antibiotics. The gene *rrsB*, expressed in the plasmid pAM552 (gift from A. Mankin’s laboratory, University of Illinois at Chicago, Chicago, IL, as in Addgene #154131), was modified to contain the A1408G mutation by amplifying the plasmid with the mutagenic primers GCA​CCA​TGG​GAG​TGG​GTT​GC and GAC​GGG​CGG​TGT​GTA​CAA​GG, phosphorylated at the 5′ end. The plasmid was then recircularized using T4 DNA ligase (Thermo Fisher Scientific) and later transformed into *E. coli* DH5α cells (Invitrogen). The mutation was confirmed by Sanger sequencing of different colonies, and the plasmid with the modified *rrsB* was then transformed into the *E. coli* strain SQ171 (gift from A. Mankin’s laboratory) with all seven rRNA deleted from the genome. The cells were made electrocompetent as in refs. 28 and 29 and aliquoted for microscopy in volumes of 20 μL stored at −80 °C.

### Electroporation of Labeled tRNA.

*E. coli* DH5α cells (Invitrogen) were previously diluted five times on 10% glycerol (OD_600_ [optical density at 600 nm] = 60 ± 10) and stored in aliquots of 20 µL at −80 °C. One aliquot of cells (SQ171, for experiments with aminoglycoside-resistant ribosomes) was mixed with 1.5 pmol of Phe-[Cy5]tRNA^Phe^ or 2 pmol of fMet-[Cy5]tRNA^fMet^ (prepared as in ref. 28) and incubated 1 min on ice. The mixture was transferred to a cold (∼4 °C) electroporation cuvette (MBP, 1 mm) and pulsed with 1.9 kV with a MicroPulser (Bio-Rad), which generated a decay time constant of 5.8 ± 0.1. Immediately after, 1 mL of EZ RDM (Teknova) + 0.2% glucose was added to the cuvette to gently resuspend the cells. The suspension was pipetted down to a culture tube and incubated for 30 min at 37 °C and shaking at 200 rpm. The cells were pelleted at RCF 2415 × g for 3 min (MiniSpin, Eppendorf) and washed three times with fresh RDM (room temperature) to remove nonelectroporated tRNA. Cells were finally resuspended in RDM (OD_600_ = 0.02) containing 1 µM SYTOX blue dead cell stain (Invitrogen).

### Microscopy Sample Preparation and Antibiotic Treatment.

The cell mixture (0.6 µL, approx. OD_600_ 0.02) was placed on a 2% agarose (SeaPlaque GTG Agarose, Lonza) pad containing 1 µM SYTOX blue in a microscope slide and incubated for 1 h at 37 °C to allow the formation of microcolonies of 2–16 cells. A solution of RDM containing 10 or 100 µg/mL of apramycin, gentamicin, or paromomycin was injected into the microscope slide to cover the agarose pad and incubated at 37 °C for 1 h to ensure antibiotic saturation before imaging. Imaging of the cells was started 60 min after antibiotic injection. Microscopy experiments were done in 2–5 replicas, each replica containing 150–350 microcolonies.

### Optical Setup.

A Nikon Eclipse Ti-E inverted microscope with a CFI Apo TIRF 100 × 1.49 NA (Nikon) objective was used. An EMCCD camera (Andor iXon 897 Ultra) equipped with a 2.0× lens (Diagnostic Instruments DD20NLT) was used to acquire bright-field and fluorescence images. An Infinity 2–5 M camera (Lumenera) was used for phase-contrast imaging. [Cy5]tRNA tracking was done using a 639-nm laser (Coherent Genesis MX 639–1000 STM) at a final power density of 5 kW/cm^2^ on the sample as stroboscopic pulses of 1.5 ms per 5 ms of camera exposure. SYTOX blue imaging was done using a 405-nm laser (Cobolt MLD) at a power density of 17 W/cm^2^ for 21 ms of camera exposure.

### Single-Molecule Tracking and HMM Analysis.

The cell segmentation, single-molecule tracking, and HMM analysis were performed as in ref. 28. *E. coli* DH5α cells were segmented using the phase-contrast images to contour the cells. Incorrectly segmented cells (e.g., truncated cell-outlines), cells with no fluorescent particles, or cells stained with SYTOX blue (dead) were manually removed. The fluorescent molecules were detected within the segmented cells by using a radial symmetry-based algorithm (48). After, the localization of the molecules and their associated uncertainty were extracted by applying a Gaussian-spot model and a maximum a posteriori fit (49). The trajectories were thereafter estimated with the uTrack algorithm (50) starting from the frame where the number of detected particles inside a cell was two or less. The trajectory building allowed gaps up to three steps.

The trajectories were then fitted to multiple models of a set number of diffusive states using an HMM algorithm (49). The quality of those models was evaluated using the AIC, the best-fitting model size was selected, and then coarse-grained to a two-state model where particles diffusing below 1 µm^2^/s were considered ribosome-bound tRNA, and molecules diffusing above the mentioned threshold were considered as free tRNA.

## Supplementary Material

Supplementary File

Supplementary File

## Data Availability

Microscopy data that support the findings of this study are available at SciLifeLab Data Repository (DOI: 10.17044/scilifelab.13741903).
