# Supplementary material for: Real-time measurements of aminoglycoside effects on protein synthesis in live cells
Source: Proc Natl Acad Sci U S A. 2021 Feb 22;118(9):e2013315118. doi: 10.1073/pnas.2013315118 (PMC7936356; doi:10.1073/pnas.2013315118)
Supplement: Supplementary File [file pnas.2013315118.sapp.pdf]

Supporting Information

Real-time measurements of aminoglycoside effects on protein synthesis in live cells

Javier Aguirre Rivera, Jimmy Larsson, Ivan L. Volkov, A. Carolin Seefeldt, Suparna Sanyal, and Magnus Johansson

Video S1. Representative video of the microfluidics growth experiments. In the video, the *E. coli* cells grow for approximately 30 min in rich-defined-media (RDM) before the antibiotic treatment. Then the media supply is changed to RDM + Apramycin (100 µg/ml), RDM + Gentamicin (100 µg/ml), or RDM + Paromomycin (100 µg/ml) for approximately 60 min. In every experiment, half of the microfluidics chip was supplied with RDM without drugs for the total duration of the experiment (appr. 8 h). After 60 min of antibiotic treatment, fresh RDM without drugs was supplied for approximately 6 h. Phase-contrast images were taken at intervals of 1 min. The playback rate of the video is 12 fps.

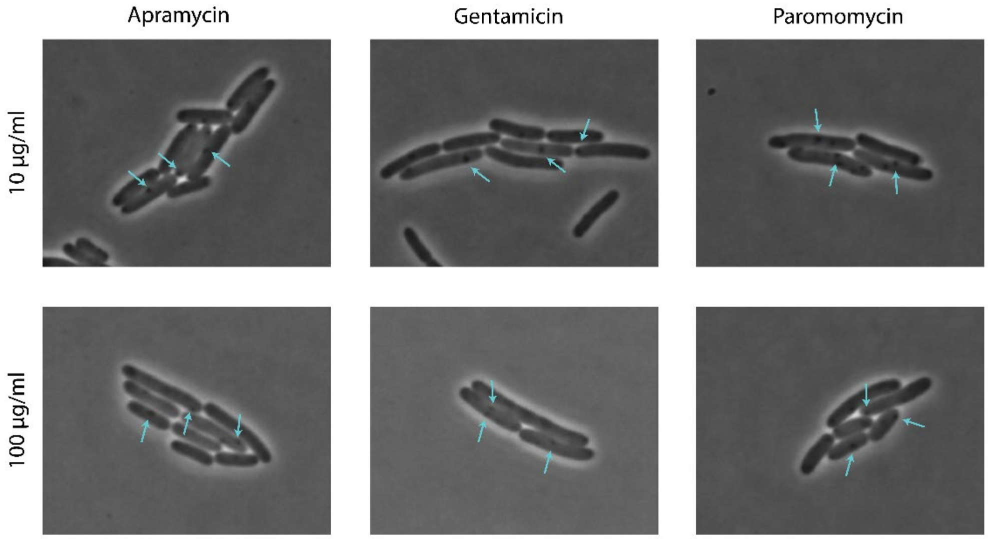

Figure S1. Exposure of *E. coli* cells to aminoglycosides induce specific cellular changes. Phase-contrast images taken after one hour of the injection of aminoglycoside antibiotics at 10 and 100 µg/ml. The arrows point to visible dark spots formed after drug treatment.

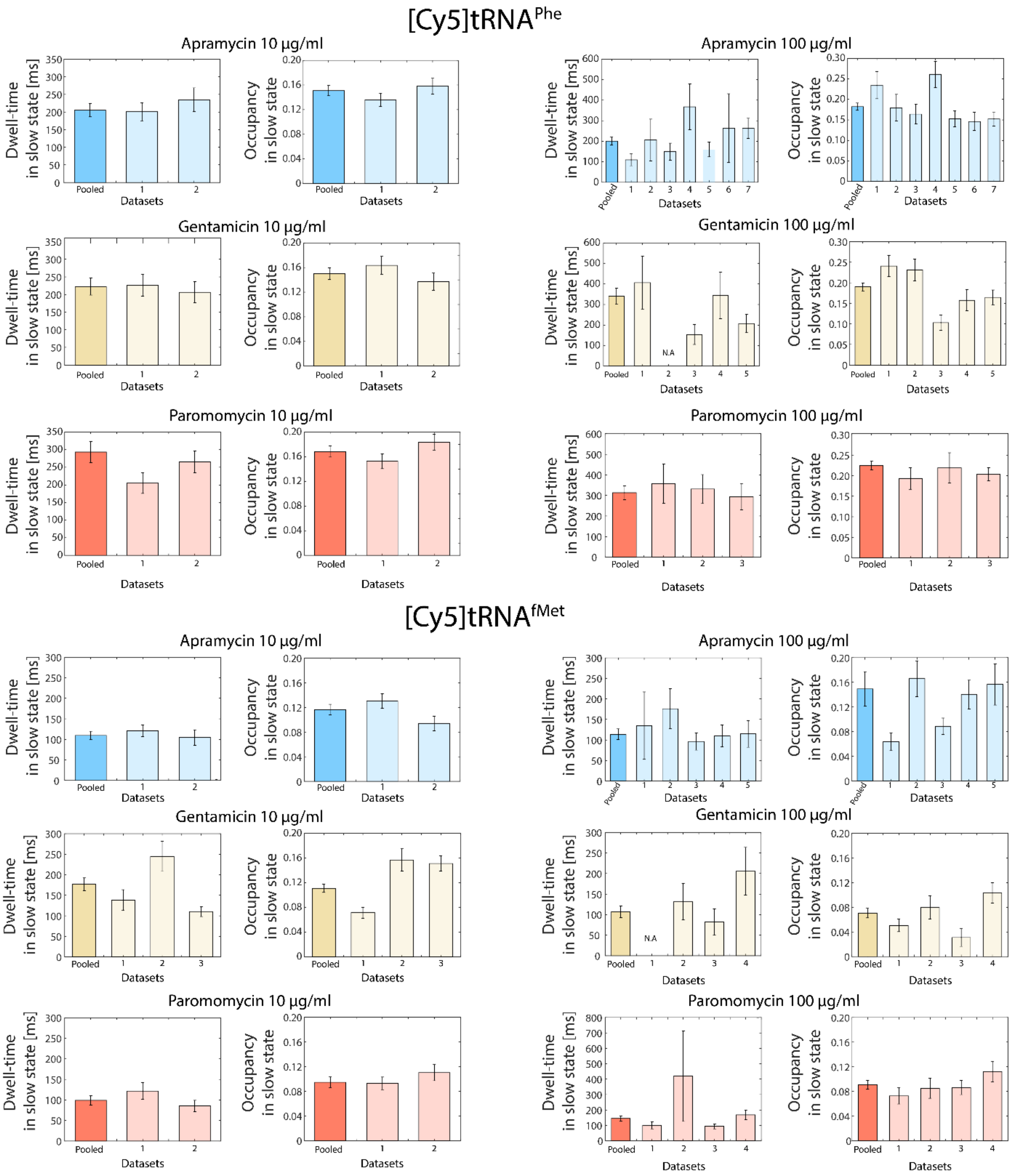

Figure S2. HMM-estimated dwell-times and occupancies at the slow diffusion state of all datasets. Datasets of [Cy5]tRNA^{Phe} and [Cy5]tRNA^{fMet} tracking in *E. coli* at 10 and 100 μg/ml of the respective aminoglycoside. In each plot, the first bar represents results generated from combining individual datasets; error bars represent bootstrap estimates of standard errors.

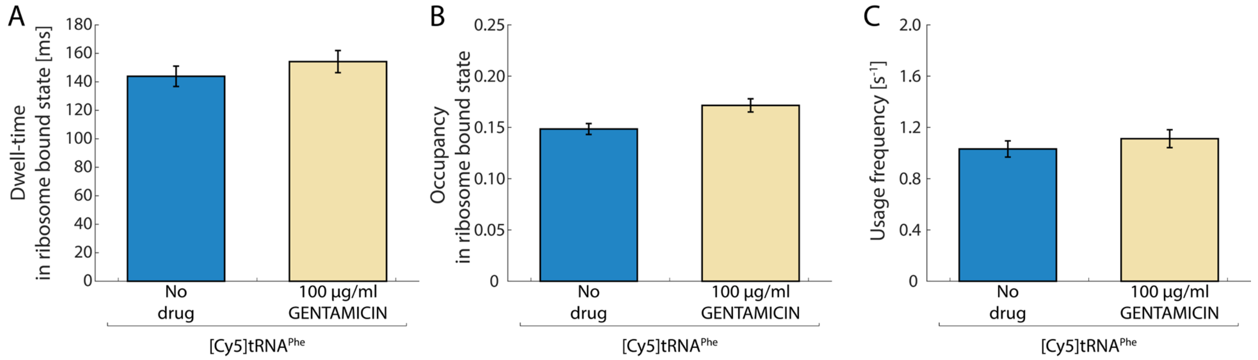

Figure S3. Effect of gentamicin on ribosome binding of [Cy5]tRNA^{Phe} in a A1408G 16S rRNA aminoglycoside resistant strain. Single-molecule tracking of Phe-[Cy5]tRNA^{Phe} in live A1408G 16S rRNA single-rRNA operon *E. coli* cells exposed to no drug or 100 µg/ml gentamicin. Error bars represent bootstrap estimates of standard errors. (a) HMM-estimated dwell-time of [Cy5]tRNA^{Phe} in the ribosome-bound state. (b) HMM-estimated occupancy of [Cy5]tRNA^{Phe} in the ribosome-bound state. (c) Usage frequency of [Cy5]tRNA^{Phe} calculated using the dwell-times and occupancies at the ribosome-bound state.

Supplementary table 1. HMM-estimated [Cy5]tRNA kinetics in E. coli DH5α cells treated with the respective aminoglycoside antibiotic. The best model size was selected using AIC. The states were coarse-grained to two states using 1 µm²/s as a threshold. The errors shown are bootstrap estimates of standard error. n = number of trajectory steps included in the analysis.

[Cy5]tRNA^{Phe}

No drug Model with lowest AIC: 9 states

	State 1			State 2			State 3			State 4			State 5			State 6			State 7			State 8			State 9		
**D (µm²/s)**	0.034	+/-	0.003	0.035	+/-	0.003	0.272	+/-	0.015	2.275	+/-	0.262	2.675	+/-	0.422	3.629	+/-	0.077	7.291	+/-	0.184	21.478	+/-	0.623	47.068	+/-	1.862
**Occupancy**	0.086	+/-	0.016	0.039	+/-	0.014	0.028	+/-	0.003	0.100	+/-	0.043	0.006	+/-	0.006	0.513	+/-	0.049	0.132	+/-	0.010	0.079	+/-	0.006	0.016	+/-	0.001
**Dwell time (s)**	0.119	+/-	0.015	0.088	+/-	0.017	0.128	+/-	0.021	0.558	+/-	NaN	0.006	+/-	0.003	0.225	+/-	0.016	4.569	+/-	4.206	1.765	+/-	1.798	0.008	+/-	0.000

	1 µm²/s					
	Slow state			Fast state		
**D (µm²/s)**	0.078	+/-	0.005	6.535	+/-	0.138
**Occupancy**	0.153	+/-	0.008	0.847	+/-	0.008
**Dwell time (s)**	0.111	+/-	0.007	0.553	+/-	0.034

Apramycin 10 µg/ml (n=42491) Model with lowest AIC: 7 states

	State 1			State 2			State 3			State 4			State 5			State 6			State 7		
**D (µm²/s)**	0.021	+/-	0.003	0.123	+/-	0.009	0.51	+/-	0.032	4.059	+/-	0.068	8.46	+/-	0.112	28.374	+/-	0.639	51.318	+/-	2.431
**Occupancy**	0.052	+/-	0.005	0.065	+/-	0.006	0.034	+/-	0.003	0.273	+/-	0.012	0.484	+/-	0.013	0.079	+/-	0.006	0.014	+/-	0.001
**Dwell time (s)**	0.298	+/-	0.058	0.289	+/-	0.057	0.09	+/-	0.01	0.723	+/-	0.103	0.431	+/-	0.054	0.472	+/-	0.161	0.007	+/-	0.000

	1 µm²/s					
	Slow state			Fast state		
**D (µm²/s)**	0.175	+/-	0.011	9.595	+/-	0.16
**Occupancy**	0.151	+/-	0.008	0.849	+/-	0.008
**Dwell time (s)**	0.205	+/-	0.019	1.005	+/-	0.083

Apramycin 100 µg/ml (n=36423) Model with lowest AIC: 9 states

	State 1			State 2			State 3			State 4			State 5			State 6			State 7			State 8			State 9		
**D (µm²/s)**	0.01	+/-	0.002	0.079	+/-	0.013	0.184	+/-	0.019	0.691	+/-	0.035	2.163	+/-	0.100	4.72	+/-	0.078	9.327	+/-	0.202	28.965	+/-	0.668	48.269	+/-	2.289
**Occupancy**	0.033	+/-	0.005	0.061	+/-	0.007	0.056	+/-	0.006	0.032	+/-	0.004	0.045	+/-	0.006	0.429	+/-	0.015	0.245	+/-	0.013	0.086	+/-	0.006	0.013	+/-	0.001
**Dwell time (s)**	0.51	+/-	0.148	2.057	+/-	NaN	0.087	+/-	0.01	0.139	+/-	0.029	0.324	+/-	0.135	0.371	+/-	0.032	4.529	+/-	3.764	0.395	+/-	0.093	0.007	+/-	0.000

	1 µm²/s					
	Slow state			Fast state		
**D (µm²/s)**	0.205	+/-	0.012	9.208	+/-	0.189
**Occupancy**	0.182	+/-	0.009	0.818	+/-	0.009
**Dwell time (s)**	0.201	+/-	0.019	0.944	+/-	0.083

Gentamicin 10 µg/ml (n=34011) Model with lowest AIC: 9 states

	State 1			State 2			State 3			State 4			State 5			State 6			State 7			State 8			State 9		
**D (µm²/s)**	0.004	+/-	0.002	0.045	+/-	0.006	0.197	+/-	0.012	0.748	+/-	0.036	2.976	+/-	0.06	7.303	+/-	0.689	8.391	+/-	0.125	27.216	+/-	0.778	57.163	+/-	2.895
**Occupancy**	0.019	+/-	0.003	0.043	+/-	0.006	0.047	+/-	0.005	0.042	+/-	0.005	0.104	+/-	0.01	0.123	+/-	0.054	0.537	+/-	0.055	0.077	+/-	0.006	0.009	+/-	0.001
**Dwell time (s)**	0.393	+/-	0.231	1.139	+/-	0.999	0.078	+/-	0.009	0.263	+/-	0.064	0.643	+/-	0.173	0.608	+/-	0.367	0.343	+/-	0.264	1.007	+/-	0.619	0.006	+/-	0.000

	1 µm²/s					
	Slow state			Fast state		
**D (µm²/s)**	0.283	+/-	0.022	9.799	+/-	0.179
**Occupancy**	0.15	+/-	0.009	0.85	+/-	0.009
**Dwell time (s)**	0.223	+/-	0.024	1.44	+/-	0.165

Gentamicin 100 µg/ml (n=27740) Model with lowest AIC: 7 states

	State 1			State 2			State 3			State 4			State 5			State 6			State 7		
**D (µm²/s)**	0.017	+/-	0.004	0.142	+/-	0.008	0.773	+/-	0.04	3.916	+/-	0.091	8.107	+/-	0.143	25.697	+/-	0.864	45.923	+/-	3.171
**Occupancy**	0.072	+/-	0.006	0.064	+/-	0.006	0.054	+/-	0.006	0.233	+/-	0.013	0.483	+/-	0.014	0.086	+/-	0.006	0.008	+/-	0.001
**Dwell time (s)**	0.304	+/-	0.061	0.349	+/-	0.084	0.161	+/-	0.032	0.382	+/-	0.049	1.149	+/-	0.491	0.18	+/-	0.075	0.008	+/-	0.001

	1 µm²/s					
	Slow state			Fast state		
**D (µm²/s)**	0.275	+/-	0.021	9.142	+/-	0.175
**Occupancy**	0.19	+/-	0.010	0.81	+/-	0.010
**Dwell time (s)**	0.34	+/-	0.039	1.216	+/-	0.132

Paromomycin 10 µg/ml (n=44848) Model with lowest AIC: 9 states

	State 1			State 2			State 3			State 4			State 5			State 6			State 7			State 8			State 9		
**D (µm²/s)**	0.014	+/-	0.003	0.129	+/-	0.014	0.158	+/-	0.058	0.798	+/-	0.026	4.064	+/-	0.174	4.312	+/-	0.151	8.677	+/-	0.118	12.284	+/-	7.434	25.778	+/-	0.551
**Occupancy**	0.028	+/-	0.004	0.032	+/-	0.019	0.049	+/-	0.019	0.059	+/-	0.005	0.084	+/-	0.014	0.043	+/-	0.014	0.557	+/-	0.012	0.005	+/-	0.002	0.143	+/-	0.006
**Dwell time (s)**	0.716	+/-	0.35	0.13	+/-	0.061	0.587	+/-	NaN	0.172	+/-	0.033	0.123	+/-	0.118	0.548	+/-	0.552	1.071	+/-	0.186	0.014	+/-	0.005	0.125	+/-	0.022

	1 µm²/s					
	Slow state			Fast state		
**D (µm²/s)**	0.352	+/-	0.02	10.936	+/-	0.159
**Occupancy**	0.168	+/-	0.009	0.832	+/-	0.009
**Dwell time (s)**	0.293	+/-	0.03	1.085	+/-	0.100

Paromomycin 100 µg/ml (n=32911) Model with lowest AIC: 9 states

	State 1			State 2			State 3			State 4			State 5			State 6			State 7			State 8			State 9		
**D (µm²/s)**	0.008	+/-	0.002	0.13	+/-	0.022	0.135	+/-	0.044	0.509	+/-	0.021	1.865	+/-	0.067	5.979	+/-	0.161	10.082	+/-	0.249	28.199	+/-	0.745	47.414	+/-	3.102
**Occupancy**	0.023	+/-	0.005	0.059	+/-	0.023	0.073	+/-	0.023	0.069	+/-	0.007	0.047	+/-	0.005	0.379	+/-	0.02	0.244	+/-	0.019	0.096	+/-	0.006	0.009	+/-	0.001
**Dwell time (s)**	3.805	+/-	1.154	0.061	+/-	0.044	2.517	+/-	NaN	0.351	+/-	0.079	0.144	+/-	0.024	0.833	+/-	0.165	2.108	+/-	1.174	0.389	+/-	0.121	0.007	+/-	0.000

	1 µm²/s					
	Slow state			Fast state		
**D (µm²/s)**	0.236	+/-	0.014	10.272	+/-	0.196
**Occupancy**	0.225	+/-	0.011	0.775	+/-	0.011
**Dwell time (s)**	0.313	+/-	0.034	0.984	+/-	0.099

A1408G Mutant (n=69789) Model with lowest AIC: 6 states

	State 1			State 2			State 3			State 4			State 5			State 6		
**D (µm²/s)**	0.041	+/-	0.003	0.141	+/-	0.007	0.613	+/-	0.030	5.729	+/-	0.074	6.738	+/-	0.520	50.598	+/-	1.334
**Occupancy**	0.083	+/-	0.004	0.043	+/-	0.003	0.023	+/-	0.003	0.654	+/-	0.032	0.177	+/-	0.031	0.020	+/-	0.001
**Dwell time (s)**	0.123	+/-	0.008	0.136	+/-	0.013	0.300	+/-	0.069	0.695	+/-	0.070	0.098	+/-	0.034	0.008	+/-	0.000

	1 µm²/s					
	Slow state			Fast state		
**D (µm²/s)**	0.158	+/-	0.010	7.017	+/-	0.058
**Occupancy**	0.148	+/-	0.005	0.852	+/-	0.005
**Dwell time (s)**	0.144	+/-	0.007	0.728	+/-	0.034

A1408 mutant + Gentamicin 100 µg/ml (n=69903) Model with lowest AIC: 7 states

	State 1			State 2			State 3			State 4			State 5			State 6			State 7		
**D (µm²/s)**	0.046	+/-	0.005	0.084	+/-	0.005	0.543	+/-	0.022	5.511	+/-	0.104	5.618	+/-	0.052	24.366	+/-	1.019	58.229	+/-	1.421
**Occupancy**	0.071	+/-	0.005	0.074	+/-	0.005	0.027	+/-	0.002	0.204	+/-	0.078	0.590	+/-	0.078	0.021	+/-	0.002	0.013	+/-	0.001
**Dwell time (s)**	0.145	+/-	0.012	0.163	+/-	0.012	0.126	+/-	0.015	0.053	+/-	0.017	0.337	+/-	0.147	0.769	+/-	0.428	0.007	+/-	0.000

	1 µm²/s					
	Slow state			Fast state		
**D (µm²/s)**	0.140	+/-	0.007	6.896	+/-	0.068
**Occupancy**	0.171	+/-	0.006	0.829	+/-	0.006
**Dwell time (s)**	0.154	+/-	0.008	0.593	+/-	0.029

[Cy5]tRNA^{fMet}

No drug (n=43576) Model with lowest AIC: 8 states

	State 1			State 2			State 3			State 4			State 5			State 6			State 7			State 8		
**D (µm²/s)**	0.078	+/-	0.007	0.521	+/-	0.036	2.618	+/-	0.107	5.711	+/-	0.316	5.763	+/-	0.130	8.928	+/-	0.315	22.572	+/-	0.673	59.245	+/-	2.978
**Occupancy**	0.044	+/-	0.004	0.014	+/-	0.003	0.064	+/-	0.007	0.018	+/-	0.007	0.525	+/-	0.023	0.217	+/-	0.020	0.110	+/-	0.008	0.008	+/-	0.001
**Dwell time (s)**	0.079	+/-	0.008	0.235	+/-	0.084	4.434	+/-	3.456	0.051	+/-	0.013	0.678	+/-	0.116	1.684	+/-	2.112	0.440	+/-	0.123	0.007	+/-	0.000

	1 µm²/s					
	Slow state			Fast state		
**D (µm²/s)**	0.187	+/-	0.020	8.694	+/-	0.129
**Occupancy**	0.058	+/-	0.005	0.942	+/-	0.005
**Dwell time (s)**	0.095	+/-	0.010	1.508	+/-	0.161

Apramycin 10 μg/ml (n=32559) Model with lowest AIC: 10 states

	State 1	State 2	State 3	State 4	State 5	State 6	State 7	State 8	State 9	State 10
**D (μm²/s)**	0.014 +/- 0.003	0.085 +/- 0.019	0.365 +/- 0.022	0.368 +/- 0.03	1.706 +/- 0.099	5.65 +/- 0.239	8.537 +/- 0.115	17.966 +/- 0.503	22.183 +/- 0.488	63.491 +/- 4.323
**Occupancy**	0.05 +/- 0.006	0.015 +/- 0.005	0.031 +/- 0.004	0.021 +/- 0.002	0.026 +/- 0.005	0.104 +/- 0.015	0.449 +/- 0.019	0.16 +/- 0.019	0.142 +/- 0.016	0.003 +/- 0.000
**Dwell time (s)**	0.249 +/- 0.052	0.486 +/- 0.347	0.132 +/- 0.03	0.035 +/- 0.002	0.462 +/- 0.187	0.236 +/- 0.037	1.206 +/- 0.235	4.286 +/- 3.794	0.058 +/- 0.013	0.007 +/- 0.001

	1 μm²/s	
	**Slow state**	**Fast state**
**D (μm²/s)**	0.179 +/- 0.016	12.095 +/- 0.205
**Occupancy**	0.116 +/- 0.009	0.884 +/- 0.009
**Dwell time (s)**	0.11 +/- 0.01	0.891 +/- 0.081

Apramycin 100 μg/ml (n=27806) Model with lowest AIC: 8 states

	State 1	State 2	State 3	State 4	State 5	State 6	State 7	State 8
**D (μm²/s)**	0.007 +/- 0.002	0.126 +/- 0.018	0.183 +/- 0.023	0.995 +/- 0.046	4.612 +/- 0.178	7.574 +/- 0.11	19.013 +/- 0.419	34.58 +/- 3.287
**Occupancy**	0.027 +/- 0.005	0.047 +/- 0.007	0.024 +/- 0.004	0.051 +/- 0.006	0.112 +/- 0.015	0.478 +/- 0.02	0.249 +/- 0.014	0.012 +/- 0.004
**Dwell time (s)**	0.461 +/- 0.253	0.283 +/- 0.085	0.037 +/- 0.004	0.088 +/- 0.009	0.214 +/- 0.037	0.839 +/- 0.117	0.95 +/- 0.461	0.013 +/- 0.006

	1 μm²/s	
	**Slow state**	**Fast state**
**D (μm²/s)**	0.413 +/- 0.146	10.906 +/- 0.341
**Occupancy**	0.149 +/- 0.028	0.851 +/- 0.028
**Dwell time (s)**	0.114 +/- 0.013	0.601 +/- 0.282

Gentamicin 10 μg/ml (n=58727) Model with lowest AIC: 8 states

	State 1	State 2	State 3	State 4	State 5	State 6	State 7	State 8
**D (μm²/s)**	0.001 +/- 0.001	0.038 +/- 0.004	0.275 +/- 0.013	1.285 +/- 0.053	5.093 +/- 0.097	9.314 +/- 0.119	21.093 +/- 0.315	31.064 +/- 1.714
**Occupancy**	0.016 +/- 0.004	0.052 +/- 0.004	0.042 +/- 0.004	0.043 +/- 0.004	0.165 +/- 0.011	0.388 +/- 0.014	0.271 +/- 0.012	0.022 +/- 0.007
**Dwell time (s)**	0.693 +/- 0.427	0.102 +/- 0.01	0.325 +/- 0.067	0.14 +/- 0.021	0.584 +/- 0.102	1.147 +/- 0.234	1.555 +/- 0.359	0.017 +/- 0.002

	1 μm²/s	
	**Slow state**	**Fast state**
**D (μm²/s)**	0.123 +/- 0.01	12.268 +/- 0.172
**Occupancy**	0.111 +/- 0.007	0.889 +/- 0.007
**Dwell time (s)**	0.177 +/- 0.016	1.159 +/- 0.084

Gentamicin 100 μg/ml (n=26287) Model with lowest AIC: 8 states

	State 1	State 2	State 3	State 4	State 5	State 6	State 7	State 8
**D (μm²/s)**	0.01 +/- 0.005	0.157 +/- 0.016	0.235 +/- 0.037	1.437 +/- 0.074	4.741 +/- 0.114	8.98 +/- 0.161	17.947 +/- 0.444	48.119 +/- 4.86
**Occupancy**	0.02 +/- 0.004	0.029 +/- 0.005	0.022 +/- 0.006	0.04 +/- 0.005	0.17 +/- 0.016	0.452 +/- 0.021	0.26 +/- 0.018	0.008 +/- 0.002
**Dwell time (s)**	0.498 +/- 0.429	0.049 +/- 0.006	0.727 +/- NaN	0.175 +/- 0.037	0.707 +/- 0.214	0.673 +/- 0.094	1.519 +/- NaN	0.018 +/- 0.006

	1 μm²/s	
	**Slow state**	**Fast state**
**D (μm²/s)**	0.14 +/- 0.015	10.709 +/- 0.221
**Occupancy**	0.07 +/- 0.008	0.93 +/- 0.008
**Dwell time (s)**	0.107 +/- 0.014	1.388 +/- 0.159

Paromomycin 10 µg/ml (n=26298) Model with lowest AIC: 8 states

	State 1			State 2			State 3			State 4			State 5			State 6			State 7			State 8		
**D (µm²/s)**	0.032	+/-	0.008	0.324	+/-	0.052	0.448	+/-	0.062	2.026	+/-	0.098	6.35	+/-	0.299	8.442	+/-	0.226	19.055	+/-	0.68	28.136	+/-	3.542
**Occupancy**	0.042	+/-	0.006	0.023	+/-	0.006	0.029	+/-	0.007	0.032	+/-	0.005	0.161	+/-	0.032	0.336	+/-	0.032	0.309	+/-	0.046	0.067	+/-	0.045
**Dwell time (s)**	0.678	+/-	0.324	0.031	+/-	0.006	0.246	+/-	NaN	0.088	+/-	0.014	0.399	+/-	0.085	2.617	+/-	2.092	1.544	+/-	0.588	0.05	+/-	0.03

	1 µm²/s					
	Slow state			Fast state		
**D (µm²/s)**	0.231	+/-	0.024	12.923	+/-	0.295
**Occupancy**	0.095	+/-	0.008	0.905	+/-	0.008
**Dwell time (s)**	0.099	+/-	0.012	0.991	+/-	0.107

Paromomycin 100 µg/ml (n=38250) Model with lowest AIC: 10 states

	State 1			State 2			State 3			State 4			State 5			State 6			State 7			State 8			State 9			State 10		
**D (µm²/s)**	0.002	+/-	0.002	0.048	+/-	0.009	0.295	+/-	0.057	0.368	+/-	0.019	1.368	+/-	0.061	4.425	+/-	0.083	7.882	+/-	0.256	9.57	+/-	0.123	22.75	+/-	0.423	50.48	+/-	4.497
**Occupancy**	0.02	+/-	0.004	0.026	+/-	0.004	0.006	+/-	0.002	0.039	+/-	0.004	0.025	+/-	0.004	0.123	+/-	0.01	0.126	+/-	0.022	0.472	+/-	0.024	0.158	+/-	0.011	0.005	+/-	0.001
**Dwell time (s)**	3.741	+/-	1.174	0.222	+/-	0.054	0.032	+/-	0.004	0.097	+/-	0.014	0.194	+/-	0.044	0.333	+/-	0.047	0.736	+/-	0.373	0.648	+/-	0.223	0.998	+/-	0.249	0.008	+/-	0.001

	1 µm²/s					
	Slow state			Fast state		
**D (µm²/s)**	0.192	+/-	0.016	10.911	+/-	0.191
**Occupancy**	0.091	+/-	0.007	0.909	+/-	0.007
**Dwell time (s)**	0.145	+/-	0.016	1.47	+/-	0.161
